# Changes in Proteolysis in Fermented Milk Produced by *Streptococcus thermophilus* in Co-Culture with *Lactobacillus plantarum* or *Bifidobacterium animalis* subsp. *lactis* During Refrigerated Storage

**DOI:** 10.3390/molecules24203699

**Published:** 2019-10-15

**Authors:** Sining Li, Shanhu Tang, Qiang He, Jiangxiao Hu, Jing Zheng

**Affiliations:** 1 College of Life Science and Technology, Southwest Minzu University, Chengdu 610041, China; ruoly123@126.com (S.L.); xx3728@126.com (J.H.); sherry0316@yeah.net (J.Z.); 2 College of Biomass Science and Engineering, Sichuan University, Chengdu 610065, China

**Keywords:** co-culture, proteolysis, free amino acid, proteolytic enzyme, fermented milk

## Abstract

Proteolysis in fermented milk, a complex and dynamic process, depends on the starter cultures used. This study aimed to evaluate the influence of *Lactobacillus plantarum* or *Bifidobacterium animalis* subsp. *lactis*, or both, co-fermented with *Streptococcus thermophilus*, on the changes in the proteolysis profile of fermented milk during 21-day storage at 4 °C, including the pH value, proteolytic degree, protease activity, aminopeptidase activity, free amino acid content, and electrophoresis performance. The results showed that the treatments with co-cultures exhibited a higher amount of free amino groups and neutral protease activity at an extracellular level, whereas lower pH values and aminopeptidase activities towards the six substrates at an intracellular level than the ones with a single-strain of *S. thermophilus* over the refrigerated storage were observed. In co-fermentation with *S. thermophilus*, *B. animalis* subsp. *lactis* did not significantly affect the concentrations of most free amino acids, while contributions of *L. plantarum* were found. Electrophoresis indicated that the mixed starters, especially the co-cultures containing *L. plantarum*, showed a stronger degradation for caseins than the pure *S. thermophilus* culture. These findings suggest that culture combinations may influence the proteolysis characteristics of the fermented products, and probiotic cultures must be carefully chosen for fermented production.

## 1. Introduction

Fermented milk is defined as the products of milk acidification by lactic acid bacteria (LAB) during metabolic fermentation. Milk provides most LAB with excellent substrates for growth, however, it can only support two to four cell generations [1], which can be explained by the milk medium that does not contain sufficient free amino acids and peptides for the reproduction of the organisms [2,3]. Consequently, LAB depend on their complex proteolytic system to hydrolyze milk proteins to release free amino groups for proliferation. It is reported that proteolytic action in milk is correlated with the processes for forming polypeptides and oligopeptides through the action of bacterial proteases [4,5], and further forming free amino acids and smaller peptides by bacterial peptidases [6].

Several studies have revealed that the proteases and peptidases liberated from LAB are active throughout fermentation and post-storage in fermented dairy products [3,7,8], and they show specificity towards particular split sites or sequences during proteolysis [9]. Particularly, aminopeptidases are thought to be of ultimately important peptidase in fermented milk, because of their capabilities to release single amino acid residues from the oligopeptides formed by extracellular protease activity [3,10]. The activities of proteolysis in fermented milk products affect not only the matrix structures and nutritional values of these products [9], but also their functional characteristics [11,12], and the peptides and amino acids liberated by the proteolytic activities are flavor compounds by themselves, or they serve as precursors for catabolic reactions [13]. It has been well-established that proteolysis in fermented milk is a major event in flavor development and texture improving through the degradation of proteins [14]. Therefore, proteolytic properties are one of the key determinants of dairy product quality.

In recent years, there has been an increasing interest in fermented milk products containing probiotic bacteria, as these probiotics are capable of colonizing in the gut and improving the health-promoting functions of fermented milk [15]. Some probiotic bacteria (such as *Lactobacillus plantarum* and *Bifidobacterium* spp.) grow poorly in milk on account of the lack of the essential proteolytic activity [16,17], and the practical approach is to culture these species in combination with *Streptococcus thermophilus* in the fermentation so as to enhance the viability of probiotic bacteria and to shorten the fermentation time [18,19]. This practice may cause interactions and changes in the proteolysis patterns when these probiotics are co-cultured with *S. thermophilus* in milk. In the past, the proteolytic activities and proteolytic enzymes of pure strains for individual *L. plantarum*, *Bifidobacterium* spp., and *S. thermophilus* have been determined and characterized extensively in fermented milk [3,20]. However, to our knowledge, no studies have been published on the effect of co-cultures of *S. thermophilus* with *L. plantarum* and/or *B. animalis* subsp. *lactis* on the proteolytic profiles in fermented milk.

Our previous study reported that the species and combinations of probiotics affect the product characteristics of fermented milk, and *S. thermophilus* in co-cultures with a ratio of 1:2 of *B. animalis* subsp. *lactis* and *L. plantarum* may have potential for the production of fermented dairy products [19]. In current study, we investigated the changes in proteolysis in fermented milk with different combinations of cultures over 21-day storage at 4 °C. The bacterial cultures were as follows: *S. thermophilus* (St), *S. thermophilus* with *B. animalis* subsp. *lactis* (StBa), *S. thermophilus* with *L. plantarum* (StLp), and *S. thermophilus* in co-cultures with 1:2 of *B. animalis* subsp. *lactis* and *L. plantarum* (StBaLp). The objective of the work was to further study the effects of the combinational use of probiotics on the proteolysis properties in fermented milk.

## 2. Results and Discussion

### 2.1. Changes in pH

The pH changes in fermented milk over the course of storage at 4 °C are shown in Table 1. All of the batches of fermented milks showed a rapid decline in pH values with the increase of storage time, which may reflect the growth of the bacteria and the accumulation of lactic acid [21]. Casarotti et al. [18] and Tian et al. [22] observed similar pH patterns in co-fermentation yogurt from *S. thermophilus* co-cultured with probiotics. This may be due to the continuous fermentation of LAB during storage [23], which may lead to the hydrolysis of milk proteins. Fermentation with *S. thermophilus* in conjunction with *B. animalis* subsp. *lactis* or *L. plantarum,* or both, significantly accelerated the pH drop of fermented milks (*p* < 0.05) compared with a single culture of *S. thermophilus* in the storage. Obviously, the StBaLp samples showed the lowest pH values over the monitored storage period.

### 2.2. Proteolytic Activity

There was a progressive increase in the amount of α-amino groups in fermented milk along with the extension of the storage time (Table 2). At the initial storage, fermented milk samples from StBaLp showed the greatest proteolytic capacity, producing the largest amount of free amino groups (0.27 mmol/L; *p* < 0.05), followed by the StLp and St samples, whereas the StBa treatments released the lowest amount of free amino groups (0.21 mmol/L). No significant difference in the proteolytic activity was observed among the co-culture samples after 7 or 14 days of refrigeration, but the proteolytic activity of the co-culture samples was higher than that of the fermented milk from single *S. thermophilus*. On the 21st day, the proteolytic activity of the StLp- and StBaLp-fermented milks was stronger than that of StBa (*p* < 0.05), and the StBa samples had a similar value to the St samples (*p* > 0.05). Shihata and Shah [3] revealed that *Bifidobacterium* spp. exhibited a lower free amino release ability compared with *S. thermophilus* and other lactobacillus. The strain of *L. plantarum* is considered to have a highly proteolytic activity in dairy systems [24,25], which is consistent with our findings in this study. In all of the cases, StBaLp-fermented milks showed the strongest proteolytic capacity.

### 2.3. Protease Activity

**Figure 1:**
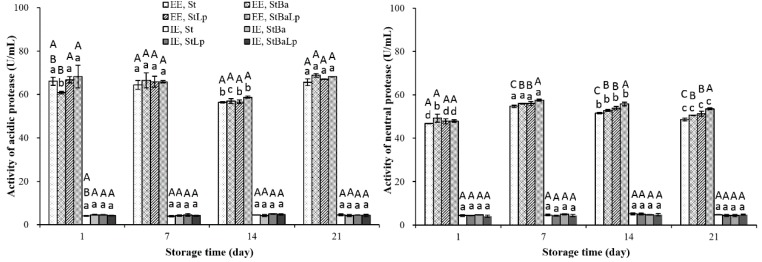
Changes in protease activity (U/mL) in fermented milk from different starter cultures during 21-day storage at 4 °C. Note: Data are means ± standard deviation (*n* = 3). Bars marked with different lower-case letters indicate significant differences among days of storage for the same batch of fermented milks (*p* < 0.05); bars marked with different upper-case letters indicate significant difference among starter cultures within the same period (*p* < 0.05).

A variety of proteases generated by LAB during milk fermentation, with a few exceptions, break down casein to large peptides [26]. As shown in Figure 1, proteases mainly located in the cell wall extracellular, and the activity in the extracellular extracts (EE), was about 10–20 times that of the intracellular extracts (IE). The acidic protease activities at the EE level in the treatments declined to minimum values on day 14 of storage, and then elevated until day 21, except for the StBa samples, which increased on the 7th and 21st day, and decreased on the 14th day, compared with the activity of the previous sampling point. The sharp increase in acidic protease activity at day 21 may be due to the proteolytic enzymes released by bacteria for their autolyzation. Our previous study also confirmed that the viability of the three strains in the fermented milks showed a drop on day 21 [19]. At the first day of cold storage, the StBa samples displayed the lowest acidic protease activity at the EE level among all of the treatments; this pattern may explain the low proteolytic activity of StBa-fermented milk under the same period (as shown in Table 2). It could be noted that the activities of the acidic protease were higher than the neutral protease at the EE level (*p* < 0.05). This is according to the previous findings of Yang et al. in probiotic soy yogurt [7]. Zakharov et al. [27] stated that most of the acidic proteases showed an optimal activity at an acid pH, as they might belong to the papain-like family of cysteine proteases.

The neutral protease activity increased in the first seven days, and then gradually decreased to the end of the storage period, which was in harmony with those obtained by Ohmiya and Sato [28]. The decrease of neutral protease activity was probably because of the inhibition of activity by acid stress caused by a low pH environment. At the last two weeks of storage, a marked difference in the activity of the neutral protease among the fermented milks at an EE level was found, and the activity of the St-fermented milk was significantly lower than that of the co-fermented milk (*p* < 0.05).

### 2.4. Aminopeptidase Activity

**Figure 2:**
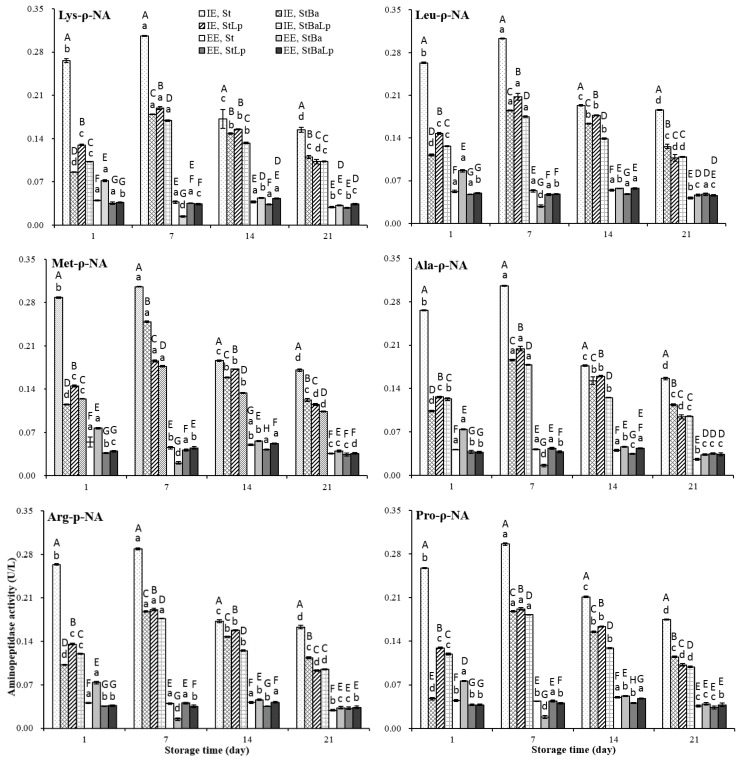
Changes in aminopeptidase activity (U/L) in fermented milk from different starter cultures during 21-day storage at 4 °C. Note: Data are means ± standard deviation (*n* = 3). Bars marked with different lower-case letters indicate significant differences among days of storage for the same batch of fermented milks (*p* < 0.05); Bars marked with different upper-case letters indicate significant difference among starter cultures within the same period (*p* < 0.05).

The activities of the aminopeptidases in the EE and IE of all of the samples over storage are plotted in Figure 2. The specific activities against the six substrates tested at the EE level were observed to be different to that at the IE level (*p* < 0.05). The aminopeptidase activity in the IE of all of the batches of fermented milks elevated to a peak at day 7 (*p* < 0.05), followed by a gradual decrease (*p* < 0.05), while the samples from different starter cultures showed a different affinity for the substrates at the EE level. Almost all of the treatments showed a higher IE activity over storage, however, an exception was also observed where the EE of the StBa-fermented milk showed a higher aminopeptidase activity than the IE for proline-containing substrates. Although it is generally accepted that most aminopeptidase are located inside the cells, the presence of some peptidases in the cell wall fraction [29], and some extracellular aminopeptidases released in the proteolytic pathway of LAB, have been detected [3,9].

The lower values at the IE level, recorded in co-culture samples, than in the St samples throughout storage were probably due to the loss of activity of aminopeptidases at the low pH values [14]. On the first day, the StLp and StBa samples demonstrated the highest and lowest affinity for the six substrates at the IE level, respectively, but the StBa samples showed the highest activity at the EE level among the co-culture samples. On day 7 of refrigeration, the aminopeptidase activity of the StBa samples towards six substrates was reduced to a minimum at the EE level. However, at the IE level, the StBa samples displayed a greater specificity towards the substrate Met-ρ-NA than that of the StLp and StBaLp samples, while towards the other five substrates it was lower than that of StLp, but higher than the StBaLp samples. It was not difficult to find that StBa-fermented milks had distinguished aminopeptidase properties from those of the two co-fermented milks in terms of their specific activity towards six substrates from day 1 to day 7, which may be related to the special nitrogen requirements of *B. animalis* subsp. *lactis* at different stages of growth [30]. After prolonged storage for 14 days, the StLp and StBaLp samples showed the highest and lowest intracellular specific activity, respectively, whereas the highest and lowest activity at the EE level was monitored in the StBa and StLp samples among the co-culture treatments. At 21 days of storage, StBa treatments presented the highest affinity against six substrates at the IE level, while StLp and StBaLp treatments did not show any significant difference towards the other four substrates, except for the derivatives of methionine and proline. The specificity towards the substrates of Met-ρ-NA and Pro-ρ-NA in the samples of StLp was higher than that of StBaLp. In this study, compared with the StBa treatments, the aminopeptidase activities in the StBaLp treatments were not significantly changed by the addition of *B. animalis* subsp. *lactis*, which indicated that *L. plantarum* had a greater influence on the substrate specificity than *B. animalis* subsp. *lactis*. Interestingly, the activity of proteolysis did not follow the activity of aminopeptidase. This observation was in accordance with an earlier report [3].

### 2.5. Free Amino Acid Content

**Figure 3:**
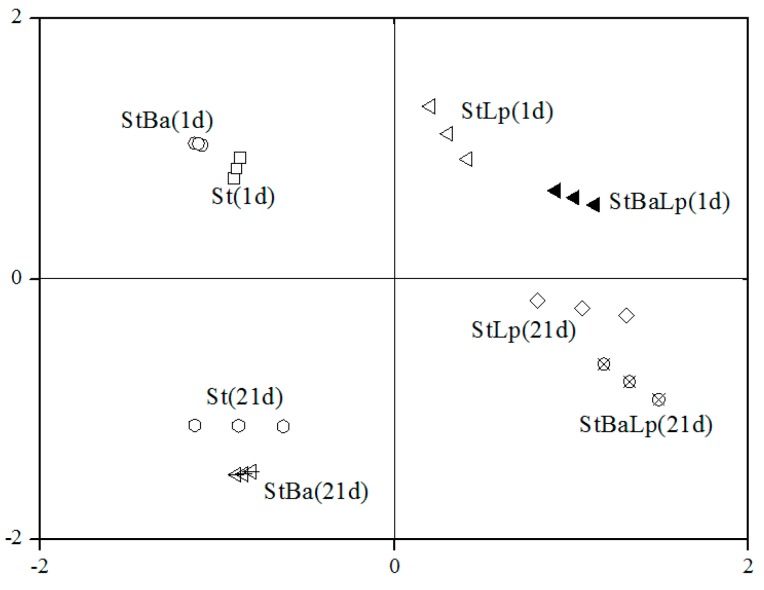
Loadings based on the principal component analysis of the free amino acid content.

The changes in free amino acids (FAAs) of the samples during storage are listed in Table 3. Initially, the total FAA concentration was 34.89 mg/kg and 36.00 mg/kg in the St and StBa samples, respectively. After 21 days, the total FAA concentration dramatically dropped to 26.02 mg/kg in the St samples, and 29.35 mg/kg in the StBa samples. It suggested that the capacity of the amino acids generated by *S. thermophilus* or *B. animalis* subsp. *lactis* was insufficient and could not meet the requirements of the bacteria at the late stage of storage. A similar trend for non-fat yoghurt over storage has been reported by Damir et al. [31]. Nevertheless, the total amounts of FAA increased to values of 39.31 mg/kg and 37.77 mg/kg from 37.68 mg/kg and 35.58 mg/kg on day 21 of storage for the StLp and StBaLp treatments, respectively. Statistically, the levels of total FAA concentrations in the co-culture samples were higher than that of the St samples (*p* > 0.05). Loadings based on the principal component analysis (PCA), in Figure 3, showed that fermented milks between StLp and StBaLp on the 21st day were positioned close to each other in the PCA diagram of PC 1 against PC 2, and the adjacent positions were found between St and StBa after 21 days storage.

**Figure 4:**
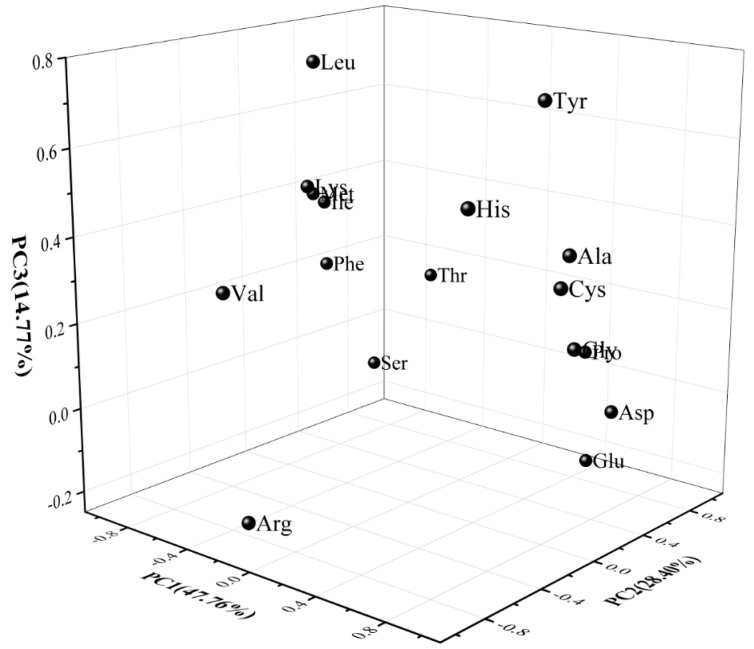
Results of the principal component analysis of the free amino acid attributes of fermented milk, showing the first three principal components (PC1, PC2 and PC3).

The PCA diagram of the FAAs data, in Figure 4, clearly explained the differences between the samples, and the relationships between attributes for the first three PCs, which were responsible for 90.33% of the variance contribution. The first PC was essentially an aspect of Gly, Cys, and Ala (positive correlation to PC 1), and Phe, Ile, and Met (negative correlation). PC 2 was Thr, Pro, and Glu (positive correlation to PC 2), and Val (negative correlation). The attributes of Leu were positively correlated with PC 3. When *B. animalis* subsp. *lactis* or *L. plantarum* was co-cultured with *S. thermophilus*, the contents of Pro, Glu, and Gly in the fermented milk could be significantly improved compared with the single *S. thermophilus*. The increase in these FAAs’ concentrations may be attributed to the biosynthesis activity of probiotics [32]. Proline was the amino acid with the highest concentrations in all of the fermented milk specimens. The results agree with the report of Yang et al. [7], who investigated fermented milks prepared from mixed cultures of *S. thermophilus*, and *L. bulgaricus* with *L. helveticus*. A large increase in the Ala concentration was observed in samples with mixed strains containing *L. plantarum*, and alanine is related to a sweet taste. The concentrations of branched-chain amino acids (Val, Leu, and Ile), which are considered to be important precursors of flavour compounds in samples without *L. plantarum,* were higher than that in the samples with *L. plantarum* during storage. The values of the sulfur-containing amino acids Met in St, and the StBa samples, was significantly higher than that in the StLp- and StBaLp-fermented milks, while for Cys, the values in the St and StBa treatments were lower than that in the StLp and StBaLp treatments. What was demonstrated here was that *L. plantarum* in co-cultures with *S. thermophilus* significantly changed the amino acid metabolism of *S. thermophilus*.

### 2.6. Electrophoresis Analysis

**Figure 5:**
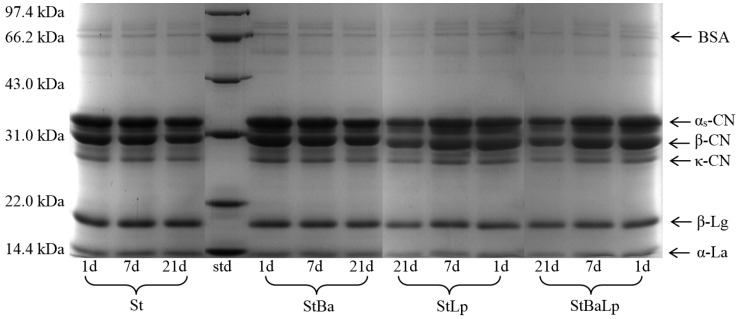
Electrophoretogram of fermented milk from different starter cultures during 21-day storage at 4 °C. Note: std (standard of molecular weight).

The changes in the protein bands of the fermented milk with different starter cultures stored for 1, 7, and 21 d at 4 °C are presented in Figure 5. As expected, all of the four fermented milks had typical milk-like protein characteristics, the most abundant proteins being α-lactalbumin (α-La), β-lactoglobulin (β-Lg), κ-casein (κ-CN), β-casein (β-CN), α-casein (α-CN), and bovine serum albumin (BSA), as confirmed by the standard proteins of the molecular weight. The band intensity of κ-CN, β-CN, and α-CN of the fermented milks visually decreased from day 1 to day 21, while the electrophoretic bands referring to α-La and β-Lg were almost unchanged in all of the treatments. This could be explained by the fact that caseins are the main substrate of the proteolytic system of LAB, which can be the correlation of the accumulation of free amino groups in the growth medium with the degradation of caseins [33,34].

As shown in Figure 5, the samples showed different proteolysis patterns depending on the starter cultures; this effect is partly due to the proteolytic activity of probiotics. A new distinguishable band near 33 kDa (range from α_s_-CN to β-CN) could be visualized in the samples with the presence of *L. plantarum,* compared with the samples with an absence of *L. plantarum*. This accounted for the intrinsically high proteolytic activity of *L. plantarum*. Ghosh et al. [35] observed that the mixture of *S. thermophilus* and *L. plantarum* had a stronger proteolysis for milk protein than the single-strain of *S. thermophilus*, and the mixed cultures gave bands beyond 10 kDa that could not be visible. After 21 days of storage, there was relatively little change in the intensities of the protein bands in the St sample, whereas obvious caseins degradation of co-fermented milk were observed throughout storage, corresponding to κ-CN, β-CN, and α_s_-CN. The analysis of the electrophoresis in this study was consistent with the results of the proteolytic activity of fermented milk.

## 3. Materials and Methods

### 3.1. Microbial Strains and Their Activation

A strain of *Streptococcus thermophilus* in a freeze-dried direct vat set form with an activity of 250 Danisco unit were kindly provided by Danisco (Kunshan, China). *Lactobacillus plantarum* (CICC-20263) and *Bifidobacterium animalis* subsp. *lactis* (CICC-21717) were collected from the China Center of Industrial Culture Collection (Beijing, China). Both *L. plantarum* and *B. animalis* subsp. *lactis* were inoculated from their stock cultures (stored at –70 °C), by giving one transfer in deMann–Rogosa–Sharpe (MRS) broth (Hopebio, Qingdao, China) at 37 °C for 24 h. After two successive transfers in MRS, these activated cultures were further transferred into a sterilized milk medium at 37 °C for 6–7 h, to reach an initial microbial concentration of 10^7^ cfu/mL for the experiment.

### 3.2. Fermented Milks Preparation

The following four bacterial cultures were conducted to ferment milk in trials: St, StBa, StLp, and StBaLp. The fermented milks were prepared according to a reported procedure [36]. Fresh milk (New Hope Diary, Chengdu, China) was heated at 90 °C for 10 min, and cooled to 43–45 °C in an ice bath. Subsequently, the milk was mixed with bacterial cultures. In all of the treatments, *S. thermophilus* were added at 0.1% (*w/v*) of milk (approximately 10^8^ cfu/mL), while *B. animalis* subsp. *lactis* and *L. plantarum* were added alone or simultaneously at an initial concentration of 10^7^ cfu/mL of milk. The mixtures were put into sterilized glass containers and incubated at 42 °C until a pH 4.60 was reached. After incubation, the fermented milk samples were stored at 4 ± 1 °C for 21 days. THe samples were analyzed at weekly intervals, up until the third week.

### 3.3. pH Determination

The fermented milk was vortexed with deionized water in 1:9 (*w/w*) before determination [37]. The changes in samples in the pH value were monitored using a pH meter (Sanxin, Shanghai, China) at room temperature (20 ± 2 °C).

### 3.4. Proteolysis Evaluation

The proteolysis activity was evaluated by measuring the free amino groups using the o-phthaldialdehyde (OPA) method of Church et al. [38]. Briefly, 2 g of fermented milk, and 1 mL of deionized water were mixed with 5 mL of 0.75 mol/L trichloroacetic acid (TCA) for 10 min, and filtered using Whatman No. 4 filter paper. Aliquots of 200 μL of the TCA filtrate and 4 mL of the OPA reagent were vortexed and reacted at room temperature (20 ± 2 °C) for 10 min. The absorbance at 340 nm was measured by a spectrophotometer (Aoyi, Shanghai, China). The free amino groups were calculated against the L-leucine standards (0–10 mmol/L).

### 3.5. Crude Enzyme Extraction

The cell wall extracellular extracts and intracellular extracts from the growth medium were prepared according to the method of Ramchandran & Shah [39]. The cells were collected from the fermented milk (10 g) by centrifugation at 12,000× *g* for 15 min (4 °C). The supernatant was harvested as the EE for the enzyme assays, while the pellet was washed twice, with 10 times the volume of 0.9% (*w/v*, NaCl) saline water, and centrifuged (5000× *g* for 15 min at 4 °C) each time so as to remove the saline water. The washed pellet was resuspended in 5 mL of 0.05 mol/L Tris-HCl buffer (pH 8.5), and sonicated at 40 KHz (Scientz Biotechnology Co., Ltd., Ningbo, China) for 10 min at 4 °C. The supernatant obtained after centrifugation (12,000× *g* for 15 min at 4 °C) was designated as the IE for the enzymatic assays.

### 3.6. Protease Assays

The protease activity in fermented milk was determined using the method of Li et al. [40]. In brief, the substrate casein (Sigma-Aldrich, St. Louis, USA) was dissolved and further diluted to a final concentration of 1% (*w/v*), with 0.05 mol/L lactate buffer (pH 3.0) and phosphate buffer (pH 7.0), respectively. The reaction system containing 1 mL of enzyme extract and 1 mL of substrate solution was incubated at 37 °C for 20 min, then the enzymatic reaction was terminated by adding 2 mL of 0.4 mol/L TCA. Subsequently, the mixtures were centrifuged at 5000× *g* for 15 min (4 °C), to obtain the supernatant. A volume of 1 mL of supernatant, 5 mL of 0.4 mol/L sodium carbonate, and 1 mL of Folin–Ciocalteu reagent (Yuanye Bio-Technology Co., Ltd., Shanghai, China) were incubated at 37 °C for 20 min. The optical densities of the reaction mixtures were read by a spectrophotometer (Aoyi, Shanghai, China) at 660 nm. The enzyme blanks were also monitored as the control. One unit of protease activity was defined as the amount of enzymes that release 1 μg of tyrosine per min per mL of casein at 37 °C.

### 3.7. Aminopeptidase Assays

An analysis of the aminopeptidase activities in fermented milk was performed using chromogenic substrates: amino acid derivate of ρ-nitroanilide (ρ-Na), namely, Lys-ρ-Na, Leu-ρ-Na, Met-ρ-Na, Ala-ρ-Na, Arg-ρ-Na, and Pro-ρ-Na, by a method reported Fernandez-Espla et al. [41]. A volume of 200 μL of enzyme extract, 800 μL of 50 mmol/L Tris-HCl buffer (pH 7.0), and 100 μL of 10 mmol/L substrate solution (Sigma-Aldrich, St. Louis, USA) were incubated at 37 °C for 20 min. The reaction was stopped by the addition of 2 mL of 30% (*v/v*) acetic acid. The released ρ-nitroanilide was monitored by measuring the absorbance at 410 nm (Aoyi, Shanghai, China). The content of ρ-nitroanilide was calculated from 9024 mol^−1^cm^−1^ of the molar absorption coefficient. One unit of enzyme activity was defined as the amount of enzyme required to release 1 μmol of ρ-nitroanilide per min per L of extract under the above assay conditions.

### 3.8. Free Amino Acid Analysis

The free amino acid contents of the fermented milk samples were evaluated by an automatic amino acid analyzer (Sykam, Eresing, Germany), equipped with a LCA K 06/Na analytical column (150 mm × 4.6 mm, 7 μm), according to the method published by Das et al. [42]. The fermented milk samples were precipitated by 5% (*w/v*) TCA and centrifuged (10,000× *g* for 20 min at 4 °C). Then, the supernatants were filtered through a 0.45 µm membrane (Jinteng, Tianjin, China) to analyze. The samples were run by a two-solvent gradient: solution A was 40 mmol/L of citric acid buffer (pH 3.45), and solution B was 70 mmol/L citric acid buffer (pH 10.85). The injection sample volume was 50 μL and the flow rate was 0.45 mL/min, and the FAAs were analyzed at 570 nm and 440 nm. By comparison with known standards for amino acids (Sigma-Aldrich, St. Louis, USA), quantitative data of the FAAs were obtained.

### 3.9. Polyacrylamide Gel Electrophoresis

The fermented milk samples were prepared for electrophoretic analysis according to Laemmli [43]. Each portion of sample (1 mg/mL) was diluted with a loading buffer and denatured in a boiling water bath for 5 min. After centrifugation at 10,000× *g* for 10 min (4 °C), the harvested proteins’ supernatant (10 µL) were loaded per lane for separating protein bands, using a 12% polyacrylamide gel with a 5% stacking gel. The separation was completed after 1.5 h at a voltage of 120 V. The gel was stained with 1g/L Coomassie brilliant blue R-250 (Sigma-Aldrich, St. Louis, USA) for 1 h, and then de-stained with a solution of 7.5% (*v/v*) methanol and 7.5% (*v/v*) acetic acid for 6 h. The gel pictures were taken using a Bio-Rad gel imager (Hercules, CA, USA). The protein concentration of the samples was measured by the Coomassie blue method [44].

### 3.10. Statistical Processing

All of the assays were performed in triplicate. The data were analyzed by the two-way analysis of variance (ANOVA) with repeated measurements for fully 4 × 4 factorial design. After indications of significant interactions of starter treatments with time-points, statistically significant differences among the treatments or storage times were processed by one-way (ANOVA) with 95% confidence intervals, and multiple comparisons were performed with Ducan’s method using the SPSS 21.0 software package (IBM, Chicago, USA). The overall difference between the samples in the free amino acids was assessed with principal components analysis.

## 4. Conclusions

The proteolytic performance of fermented milk produced by *S. thermophilus* in co-cultures with *B. animalis* subsp. *lactis* or *L. plantarum* was modified during the 21 days of refrigerated storage. All of the fermented milks exhibited increasing proteolysis with extended time, with both of the StLp and StBaLp treatments showing the highest proteolytic activity. An analysis of the activity of the protease and aminopeptidase showed that the co-cultures treatments had a higher neutral protease activity at the EE level, but a lower aminopeptidase activity at the IE level than the St treatment during storage. The free amino acid profiles for the StLp and StBaLp treatments were quite similar, whereas the profile for the St samples was similar to those of the StBa samples. The SDS-PAGE analysis indicated that co-cultures containing *L. plantarum* promoted the hydrolysis of caseins. This study suggests that *L. plantarum* has a significantly larger effect than *B. animalis* subsp. *lactis* on the proteolysis of fermented milk produced by co-fermentation with *S. thermophilus* during chilled storage.

## Figures and Tables

**Table 1:** Changes in pH in fermented milk from different starter cultures during 21-day storage at 4 °C.

Starter Cultures	Day 1	Day 7	Day 14	Day 21
St	4.38 ± 0.01 ^aA^	4.27 ± 0.01 ^bA^	4.21 ± 0.01 ^cA^	4.19 ± 0.00 ^dA^
StBa	4.32 ± 0.01 ^aB^	4.23 ± 0.01 ^bB^	4.17 ± 0.01 ^cB^	4.14 ± 0.01 ^dB^
StLp	4.30 ± 0.02 ^aB^	4.21 ± 0.01 ^bBC^	4.15 ± 0.01 ^cBC^	4.11 ± 0.01 ^dC^
StBaLp	4.30 ± 0.01 ^aB^	4.19 ± 0.01 ^bC^	4.14 ± 0.01 ^cC^	4.10 ± 0.01 ^dC^

Note: Data are means ± standard deviation (*n* = 3). Different lower-case letters in the same row indicate significant differences among days of storage (*p* < 0.05); different upper-case letters in the same column indicate significant differences among starters (*p* < 0.05). St—*S. thermophilus*; StBa—*S. thermophilus* with *B. animalis* subsp. *lactis*; StLp—*S. thermophilus* with *L. plantarum*; StBaLp—*S. thermophilus* in co-cultures with 1:2 of *B. animalis* subsp. *lactis* and *L. plantarum*.

**Table 2:** The proteolytic activity (mmol/L) of the various combinations of starter cultures in fermented milk during 21-day storage at 4 °C.

Starter Cultures	Day 1	Day 7	Day 14	Day 21
St	0.23 ± 0.01 ^dBC^	0.37 ± 0.01 ^cB^	0.49 ± 0.00 ^bB^	0.51 ± 0.01 ^aB^
StBa	0.21 ± 0.02 ^cC^	0.39 ± 0.02 ^bA^	0.52 ± 0.01 ^aA^	0.53 ± 0.02 ^aB^
StLp	0.25 ± 0.01 ^dB^	0.41 ± 0.01 ^cA^	0.52 ± 0.01 ^bA^	0.55 ± 0.01 ^aA^
StBaLp	0.27 ± 0.01 ^dA^	0.41 ± 0.01 ^cA^	0.53 ± 0.01 ^bA^	0.56 ± 0.00 ^aA^

Note: Data are means ± standard deviation (*n* = 3). Different lower-case letters in the same row indicate significant differences among days of storage (*p* < 0.05); Different upper-case letters in the same column indicate significant differences among starters (*p* < 0.05).

**Table 3:** Changes of free amino acids (mg/kg) in fermented milk from different starter cultures during 21-day storage at 4 °C.

Amino Acids	St Treatment			StBa Treatment			StLp Treatment			StBaLp Treatment		
	Day 1	Day 21	Increment	Day 1	Day 21	Increment	Day 1	Day 21	Increment	Day 1	Day 21	Increment
Asp	1.94 ± 0.01 ^c^	1.14 ± 0.09 ^d^	−0.80	1.92 ± 0.02 ^c^	1.37 ± 0.05 ^d^	−0.55	2.52 ± 0.08 ^ab^	2.26 ± 0.35 ^bc^	−0.26	2.68 ± 0.19 ^a^	2.46 ± 0.17 ^ab^	−0.22
Thr	1.91 ± 0.06 ^ab^	1.21 ± 0.11 ^c^	−0.70	2.11 ± 0.03 ^a^	1.13 ± 0.04 ^cd^	−0.98	1.78 ± 0.18 ^b^	1.30 ± 0.22 ^c^	−0.48	1.30 ± 0.05 ^c^	0.84 ± 0.19 ^d^	−0.46
Ser	0.85 ± 0.00 ^ab^	0.70 ± 0.03 ^bc^	−0.15	0.95 ± 0.03 ^a^	0.60 ± 0.06 ^cd^	−0.35	0.73 ± 0.10 ^bc^	0.48 ± 0.11 ^de^	−0.25	0.64 ± 0.04 ^c^	0.44 ± 0.08 ^e^	−0.20
Glu	2.32 ± 0.07 ^cd^	1.38 ± 0.17 ^f^	−0.94	2.38 ± 0.02 ^bc^	1.15 ± 0.06 ^g^	−1.23	2.60 ± 0.09 ^b^	2.12 ± 0.14 ^de^	−0.48	3.11 ± 0.05 ^a^	2.07 ± 0.09 ^e^	−1.04
Gly	0.06 ± 0.00 ^d^	0.14 ± 0.11 ^d^	0.08	0.09 ± 0.01 ^d^	0.17 ± 0.02 ^d^	0.08	1.09 ± 0.02 ^bc^	1.73 ± 0.21 ^a^	0.64	1.06 ± 0.02 ^c^	1.33 ± 0.18 ^b^	0.27
Ala	4.05 ± 0.14 ^bcd^	3.81 ± 0.76 ^cd^	−0.24	3.97 ± 0.05 ^cd^	3.57 ± 0.03 ^d^	−0.40	4.76 ± 0.13 ^b^	5.79 ± 0.15 ^a^	1.03	4.56 ± 0.16 ^bc^	5.61 ± 0.33 ^a^	1.05
Cys	1.21 ± 0.01 ^cd^	1.00 ± 0.22 ^d^	−0.21	1.16 ± 0.06 ^cd^	1.10 ± 0.11 ^d^	−0.06	1.40 ± 0.14 ^bc^	1.98 ± 0.02 ^a^	0.58	1.61 ± 0.07 ^b^	2.20 ± 0.13 ^a^	0.59
Met	1.20 ± 0.03 ^ab^	0.93 ± 0.11 ^bc^	−0.27	1.26 ± 0.08 ^a^	1.10 ± 0.04 ^abc^	−0.16	1.10 ± 0.18 ^abc^	0.83 ± 0.13 ^cd^	−0.27	0.41 ± 0.02 ^e^	0.60 ± 0.21 ^de^	0.19
Val	0.22 ± 0.01 ^c^	0.30 ± 0.07 ^b^	0.08	0.20 ± 0.01 ^c^	0.42 ± 0.02 ^a^	0.22	0.07 ± 0.01 ^d^	0.23 ± 0.04 ^bc^	0.16	0.07 ± 0.01 ^d^	0.26 ± 0.04 ^bc^	0.19
Ile	1.40 ± 0.04 ^a^	0.66 ± 0.04 ^cd^	−0.74	1.54 ± 0.01 ^a^	1.06 ± 0.01 ^b^	−0.48	0.86 ± 0.20 ^bc^	0.52 ± 0.16 ^de^	−0.34	0.18 ± 0.01 ^f^	0.42 ± 0.06 ^e^	0.24
Leu	0.50 ± 0.01 ^a^	0.31 ± 0.02 ^bc^	−0.19	0.57 ± 0.01 ^a^	0.48 ± 0.07 ^ab^	−0.09	0.27 ± 0.09 ^cd^	0.41 ± 0.13 ^abc^	0.14	0.12 ± 0.00 ^d^	0.41 ± 0.12 ^abc^	0.29
Tyr	0.19 ± 0.01 ^cd^	0.04 ± 0.01 ^e^	−0.15	0.18 ± 0.00 ^cd^	0.03 ± 0.01 ^e^	−0.15	0.26 ± 0.08 ^c^	0.51 ± 0.06 ^a^	0.25	0.11 ± 0.01 ^de^	0.38 ± 0.04 ^b^	0.27
Phe	1.15 ± 0.01 ^a^	0.92 ± 0.26 ^a^	−0.23	1.18 ± 0.01 ^a^	0.89 ± 0.11 ^a^	−0.29	0.92 ± 0.07 ^a^	0.71 ± 0.17 ^a^	−0.21	0.61 ± 0.22 ^a^	0.54 ± 0.12 ^a^	−0.07
Lys	1.40 ± 0.00 ^ab^	1.51 ± 0.05 ^a^	0.11	1.42 ± 0.06 ^ab^	1.47 ± 0.02 ^a^	0.05	0.93 ± 0.05 ^d^	1.35 ± 0.09 ^b^	0.42	0.92 ± 0.05 ^d^	1.06 ± 0.06 ^c^	0.14
His	4.73 ± 0.06 ^c^	4.67 ± 0.54 ^c^	−0.06	4.52 ± 0.11 ^c^	5.67 ± 0.47 ^b^	1.15	5.03 ± 0.04 ^bc^	7.46 ± 0.30 ^a^	2.43	4.87 ± 0.21 ^c^	7.85 ± 0.04 ^a^	2.98
Arg	0.75 ± 0.08 ^d^	1.34 ± 0.05 ^b^	0.59	0.91 ± 0.08 ^cd^	1.65 ± 0.11 ^a^	0.74	0.84 ± 0.01 ^cd^	0.95 ± 0.06 ^c^	0.11	0.97 ± 0.10 ^c^	1.17 ± 0.03 ^b^	0.20
Pro	11.04 ± 0.12 ^cd^	6.02 ± 0.95 ^f^	−5.02	11.67 ± 0.06 ^bc^	7.52 ± 0.08 ^e^	−4.15	12.57 ± 0.35 ^a^	10.71 ± 0.01 ^d^	−1.86	12.40 ± 0.11 ^ab^	10.19 ± 0.04 ^d^	−2.21
Total	34.89 ± 0.04 ^d^	26.02 ± 1.22 ^f^	−8.87	36.00 ± 0.12 ^bcd^	29.35 ± 1.11 ^e^	−6.65	37.68 ± 1.13 ^abc^	39.31 ± 0.76 ^a^	1.63	35.58 ± 0.35 ^bcd^	37.77 ± 1.26 ^ab^	2.19

Note: Data are means ± standard deviation (*n* = 3). Different lower-case letters in the same row indicate significant differences among days of storage (*p* < 0.05).
